# Clinical validation of a fecal PCR assay for *Helicobacter pylori* detection: multicenter comparative study with urea breath test and commercial molecular diagnostics

**DOI:** 10.1128/jcm.01762-25

**Published:** 2026-07-27

**Authors:** Yingxin Wang, Chengzhi He, Yuanxi Jiang, Zhendong Yao, Ting Jiang, Xiangwu Ding, Wensheng Pan, Boneng Mao, Changqing Yang

**Affiliations:** 1 Department of Gastroenterology and Hepatology, Tongji Hospital, School of Medicine, Tongji University 12476, Shanghai, China; 2 Department of Gastroenterology, Yixing People’s Hospital, Yixing, Jiangsu, China; 3 CellRun(Shanghai) Medical Technology Co., LTD, Shanghai, China; 4 Department of Gastroenterology, Wuhan Fourth Hospital 74497, Wuhan, Hubei, China; 5 Center for General Practice Medicine, Department of Gastroenterology, Zhejiang Provincial People’s Hospital (Affiliated People’s Hospital), Hangzhou Medical College, Hangzhou, Zhejiang, China.; Maine Medical Center Department of Medicine, Portland, Maine, USA

**Keywords:** *Helicobacter pylori*, PCR assay, urea breath test, clinical trial, sensitivity, specificity

## Abstract

*Helicobacter pylori* infection is linked to gastritis, peptic ulcers, and gastric cancer. The urea breath test (UBT), while gold standard for active infection diagnosis, poses radioactive risks and equipment dependency. Fecal molecular detection offers a non-invasive alternative but requires validation. This multicenter study evaluates a novel fecal PCR assay (Cellrun PCR assay, 16S rRNA-targeted) against UBT and a commercial kit (comparison reagents). A multicenter trial enrolled 866 patients (May 2023–Sep 2024) from four hospitals. Of 777 valid samples, positive detection rates, diagnostic consistency (kappa), and clinical performance (sensitivity/specificity) were compared across UBT, Cellrun PCR assay, and comparison reagents. Positive rates were 44.02% (UBT), 43.89% (Cellrun PCR assay), and 44.14% (comparison reagents), with no significant differences (*P* > 0.05). Cellrun PCR assay exhibited near-perfect agreement with UBT (Kappa = 0.9713, *P* < 0.001) and matched comparison reagents’ consistency (Kappa = 0.966, *P* < 0.001). Sensitivity and specificity for Cellrun PCR assay were 98.2% (AUC = 0.985), comparable to comparison reagents (AUC = 0.983; *P* = 0.614). Weak-positive agreement was 85.7%, while post-treatment sensitivity/specificity reached 94.74%/97.06% (AUC = 0.985). Cellrun PCR assay achieved 100% accuracy in interference samples (Kappa = 1.0). False negatives correlated with low bacterial loads or processing variability; false positives arose near detection thresholds. Cellrun PCR assay demonstrates UBT-comparable sensitivity and specificity, validating its equivalence to established molecular diagnostics for *Helicobacter pylori* detection.

**IMPORTANCE:**

*Helicobacter pylori* infection is a major global health burden, linked to gastritis, peptic ulcers, and gastric cancer. Current gold-standard urea breath tests (UBTs) carry radioactive risks and require specialized equipment, limiting accessibility. Fecal molecular detection offers a non-invasive alternative, but existing assays need robust validation. Our study validates the novel Cellrun PCR assay, showing it matches UBT’s accuracy and established commercial kits. This user-friendly, non-radioactive tool enhances diagnostic accessibility, especially in resource-limited settings, enabling early *H. pylori* detection and targeted treatment—critical for reducing associated morbidity and mortality. Its reliability supports broader clinical adoption, advancing global efforts to manage this prevalent infection.

## INTRODUCTION

*Helicobacter pylori* is a Gram-negative, microaerophilic, spiral-shaped pathogenic bacterium that predominantly colonizes the gastric mucosa in humans, instigating a range of upper gastrointestinal disorders, including chronic gastritis, peptic ulcers, and gastric mucosa-associated lymphoid tissue (MALT) lymphoma (1–3). The World Health Organization (WHO) has categorized *H. pylori* as a Group I carcinogen (4, 5). The global prevalence of Hp infection is reaching up to 59.2%, with particularly elevated rates in developing nations, where it can exceed 80% in certain regions, such as Africa (6–8). Transmission primarily occurs via fecal-oral and oral-oral routes, with inadequate sanitation in developing countries exacerbating the spread of this infection (9, 10). Although the majority of infected individuals remain asymptomatic, chronic infection is significantly linked to an increased risk of gastric cancer (11, 12), underscoring the necessity for early and precise diagnosis to facilitate effective disease management and prevention.

Current diagnostic modalities can be delineated into invasive and non-invasive techniques. Invasive methods, including histological examination of gastric mucosal biopsies, rapid urease tests (RUTs), and bacterial cultures, are regarded as the gold standard. However, these approaches necessitate endoscopic procedures, are inherently invasive, and are prone to sampling errors, particularly in scenarios involving low bacterial loads or morphological variations (such as coccoid forms), which can markedly diminish sensitivity (13–15). Among non-invasive diagnostic modalities, the urea breath test (UBT) is highly regarded for its exceptional sensitivity and specificity. Nonetheless, the radioactive nature of the ^14^C-UBT imposes limitations on its use in pediatric and pregnant populations, while the ^13^C-UBT is often hindered by its elevated cost. Additionally, UBT is not devoid of shortcomings, including the potential for false-negative results, particularly in scenarios characterized by low infection intensity or delayed gastric emptying, which may compromise the accurate detection of *Helicobacter pylori*. Furthermore, the administration of certain medications, such as proton pump inhibitors and antibiotics, can adversely influence test results, thereby reducing their reliability in specific clinical contexts (16–19). Serological tests, although straightforward to administer, fail to distinguish between active and past infections due to the persistence of antibodies for 6 to 8 months following eradication (20, 21). Fecal antigen detection is suitable for large-scale screening but may be compromised by antigen stability and antibody affinity, leading to potential false-negative results (22, 23). Advancements in molecular diagnostic technologies have established fecal PCR testing as a compelling method for diagnosing *Helicobacter pylori*. This technique not only allows for species-specific identification through DNA analysis but also facilitates the simultaneous detection of clarithromycin resistance mutations, notably the A2143G point mutation in the 23S rRNA gene. In comparison to the urea breath test (UBT), fecal PCR achieves a detection rate exceeding 90% for low bacterial loads (<10^3^ CFU/g) and coccoid forms, effectively bypassing the reliance on viable bacteria required by traditional culture methods (24, 25). As a non-invasive specimen, fecal samples can concurrently reflect the colonization status of both the stomach and oral cavity, thus providing substantial value for monitoring recurrence after eradication; studies suggest that combined local oral treatments can significantly improve eradication success rates (26, 27). Although current guidelines have not yet integrated fecal PCR, its potential for large-scale screening—especially among children, the elderly, and individuals contraindicated for endoscopic procedures—has garnered recognition from researchers in several countries (28–31). There is an urgent need to develop standardized operational protocols through multicenter studies and to perform methodological comparisons with commercial kits, which will be crucial for advancing its clinical application. This study aims to systematically evaluate the diagnostic performance of the fecal PCR assay through a multicenter clinical trial, utilizing UBT as a reference standard and comparing it with an already marketed product (NMPA 20223401755, Jiangsu CWBIO Century Biotechnology Co., Ltd.) across dimensions, such as sensitivity, specificity, and positive/negative agreement rates to validate its clinical applicability.

## MATERIALS AND METHODS

### Subject selection

**Fig 1:**
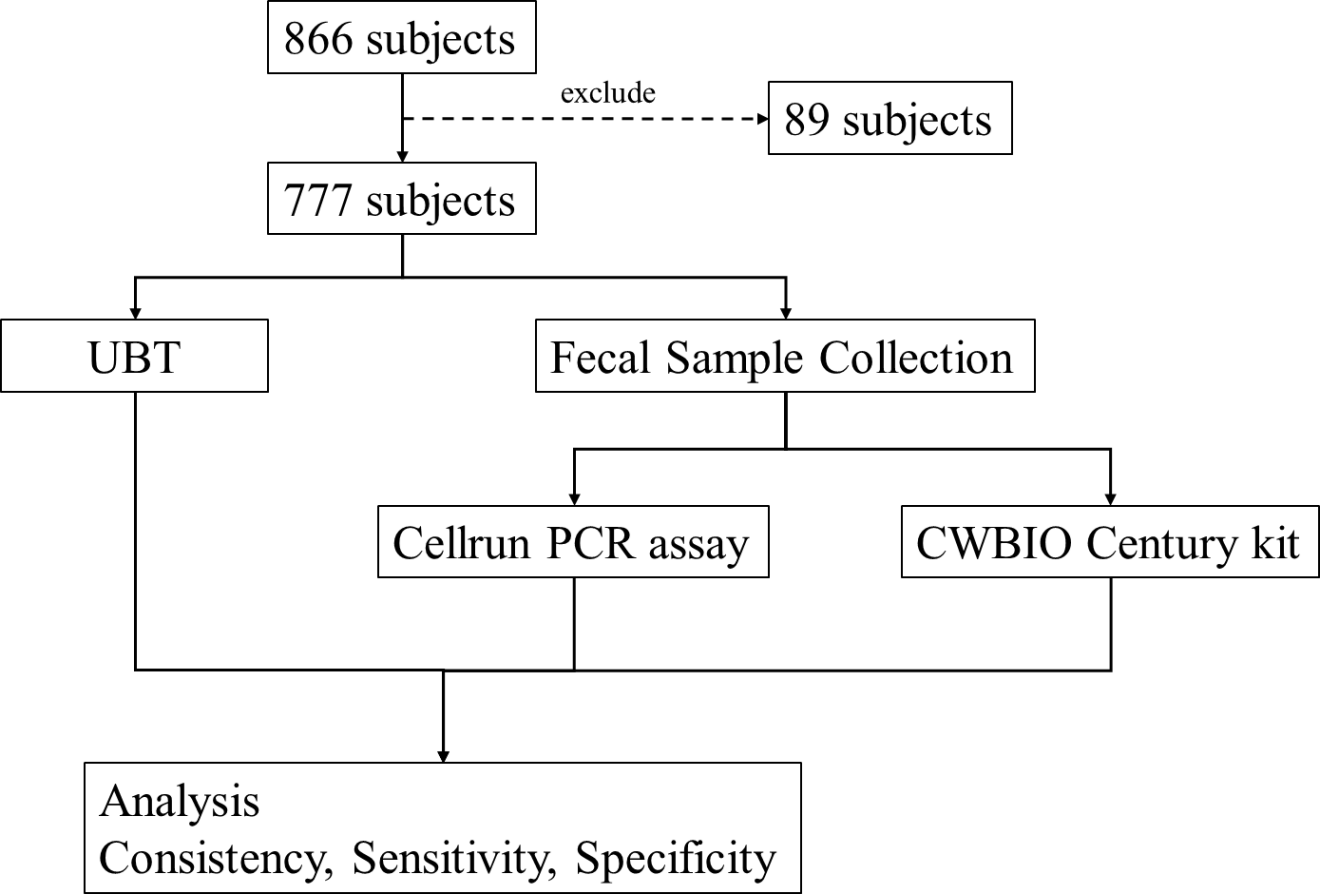
The workflow of the study.

This study represents a multicenter cross-sectional observational investigation conducted between May 2023 and September 2024 at Tongji Hospital in Shanghai (the lead institution), Zhejiang Provincial People’s Hospital, Wuhan Fourth Hospital, and Yixing People’s Hospital. A total of subjects aged 18 to 75 years who met at least one of the following criteria were included: (i) individuals exhibiting symptoms of gastric diseases, such as gastritis, peptic ulcers, or dyspepsia; (ii) asymptomatic patients with a clinical suspicion of *Helicobacter pylori* infection; (3) individuals who had not used antibiotics, bismuth compounds, proton pump inhibitors, or antimicrobial traditional Chinese medicine within 2 weeks prior to enrollment; (iv) patients who underwent *H. pylori* eradication therapy and had a time interval of more than one month since treatment. Exclusion criteria included: (i) incomplete or untraceable sample information, including demographic and clinical diagnostic background; (ii) insufficient sample size to complete the study; (iii) non-compliance with sample collection, processing, transportation, and preservation requirements as per the instructions; (iv) duplicate samples from the same provider; (v) samples deemed unsuitable for inclusion by the researchers. The study comprised two comparative groups: the experimental group employed an *in vitro* diagnostic reagent (Cellrun PCR assay) utilizing the PCR method, while the comparator group (comparison reagents) utilized a commercially available nucleic acid detection kit for *Helicobacter pylori*, specifically a PCR fluorescence probe method (National Medical Device Approval No. 20223401755, Jiangsu CWBIO Century Biotechnology Co., Ltd.) (Fig. 1).

### Fecal sample collection

All participants utilized a standardized fecal collection device for sample acquisition. The assessment reagent employed fecal sampling tubes produced by Cellrun Medical Technology Co., Ltd. (Shanghai), which included a specialized sampling spoon. Participants were instructed to collect approximately 1 mL of fresh fecal sample up to the designated mark on the tube, ensuring the spoon was fully inserted before sealing the cap tightly. Comparison reagents utilized fecal collection and preservation tubes registered by Jiangsu CWBIO Century Biotechnology Co., Ltd. (registration number Su Tai Xie Bei 20190177), where participants collected 50–100 mg fecal samples into tubes containing preservation solution, as per the instructions. Following collection, precise documentation of collection time, participant ID, and clinical diagnostic information was maintained. Assessment reagent samples were stored and transported at 2°C–37°C (≤7 days), while Comparison Reagents samples were maintained at the same temperature (≤15 days). For patients post-eradication therapy, sample collection was required to occur more than 1 month after treatment. Sample processing adhered strictly to a double-blind principle, with testing personnel blinded to UBT results and clinical diagnostic information.

### DNA extraction

DNA extraction from fecal samples was performed using a commercial nucleic acid extraction kit combined with an optimized mechanical lysis protocol. For the experimental group samples (collected using Cellrun fecal sampling devices), 200 μL of fecal suspension was added to a lysis tube containing 1.0-mm zirconium silicate beads, followed by the addition of 20 μL of proteinase K (60 mAU/mL) and 580 μL of ASL buffer. The mixture was vortexed for 5 min (3,000 rpm) and then incubated at 70°C for 10 min, with vortexing every 2 min for 30 s. Subsequently, 600 μL of absolute ethanol was added to precipitate impurities, and the mixture was centrifuged at 12,000 × *g* for 3 min. The supernatant was purified using a column (QIAamp DNA Stool Mini Kit, Qiagen). For the comparator group samples (collected using CWBIO Century preservation tubes), 500 μL of preservation solution was extracted according to the manufacturer’s instructions for biphasic lysis: first, 100 μL of lysozyme (50 mg/mL) was added and incubated at 37°C with shaking for 30 min, followed by the addition of 20 μL of mutanolysin (5,000 U/mL) and 10 μL of achromopeptidase (2,000 U/mL) for secondary enzymatic digestion.

The DNA extraction process was subjected to dual quality control: (i) the human β-actin gene was utilized as an internal control, with amplification efficiency required to be between 90% and 110%; (ii) each batch included negative controls (nuclease-free water) and positive controls (*H. pylori* ATCC 43504 standard strain). To address potential inhibitory substances in the fecal samples, a 260/280 nm absorbance ratio (acceptable range 1.7–2.0) was combined with Synthetic Positive Universal DNA (SPUD) assay for detection. If the Ct value deviated by more than two cycles, an additional treatment with 10% BSA was applied. Extracted DNA was stored at −80°C, and PCR detection was completed within 72 h to prevent degradation due to repeated freeze-thaw cycles.

### Polymerase chain reaction (PCR)

This experiment employed dual fluorescent quantitative PCR technology to simultaneously detect the 16S rRNA gene of *H. pylori* and the human β-actin internal control gene. The reaction mixture consisted of: 10 μL of 2× TaqMan Universal PCR Master Mix (including UDG enzyme for contamination prevention), 0.8 μL of each 10 μM specific primer (16S rRNA-F: 5′-GCTAAGAGATCAGCCTATGTCC-3′; 16S rRNA-R: 5′-TGGCAATCAGCGTCAGGTAAT-3′), 0.4 μL of 10 μM dual-labeled probe (16S rRNA probe: 5′-VIC-CCACACCCTAGACGGGTTA-MGB-3′; β-actin probe: 5′-CY5-TGACCGAGCGTGGCTACAGC-TAMRA-3′), 5 μL of DNA template, and nuclease-free water to a final volume of 20 μL.

The amplification program was set as follows: 2 min at 50°C for UDG enzyme pre-treatment; 10 min at 95°C for initial denaturation; followed by 45 cycles of amplification (15 s at 95°C for denaturation, and 60 s at 60°C for annealing/extension). Detection was performed using an ABI 7500 real-time fluorescence quantitative PCR instrument, with fluorescence signals collected during the extension phase of each cycle. Quality control measures included three wells of negative control (no template control) and three wells of positive control (containing 10^3^ copies/μL recombinant plasmid) for each batch.

Based on the results of the reagent kit development stage, the *H. pylori* detection cut-off Ct value for this kit was established at 39.24. A result is considered positive when the Ct value is ≤39.24. Each run requires three controls, and all quality control criteria must be met for the run to be valid. Negative Control: VIC channel was no amplification, and CY5 channel was S-shaped curve with Ct ≤ 29; Weak Positive Control: VIC channel: S-shaped curve with Ct ≤ 33; CY5 channel was S-shaped curve with Ct ≤ 29; Strong Positive Control: Both VIC and CY5 channels were S-shaped curves with Ct ≤ 29. If these conditions are not simultaneously satisfied, the PCR run is invalid and must be repeated. The criteria for result interpretation were a CT value of the 16S rRNA gene ≤37, and a CT value of the β-actin gene ≤35 indicated positivity; a CT value of the 16S rRNA gene >37 and a CT value of the β-actin gene ≤35 required retesting; a CT value of the β-actin gene >35 indicated suboptimal nucleic acid extraction. All weak-positive samples (CT values 37–42) were confirmed through repeat testing.

### Urea breath test (UBT)

All participants were required to fast for at least 4 h prior to sampling, with the carbon-13 urea breath test (^13^C-UBT) employed as the clinical reference standard. Detection utilized the HCBT-01 breath analyzer (Shenzhen Haidewei Biotechnology Co., Ltd.) and accompanying urea [^13^C] capsules (registration number H20074011), strictly following standardized operating procedures as outlined in the “Consensus on the Management of *Helicobacter pylori* Infection”: participants ingested a capsule containing 75 mg of urea [^13^C] and remained seated for 30 min, after which baseline (0 min) and post-ingestion (30 min) breath samples were collected into specialized collection bags. Samples were automatically analyzed by the instrument, with a Δ value (DOB value) ≥4.0‰ classified as positive, <2.5‰ as negative, and 2.5‰ ≤ Δ value < 4.0‰ defined as a gray area requiring retesting.

Each batch of testing included positive controls (samples from known *H. pylori*-infected patients) and negative controls (samples from healthy volunteers), with a quality control compliance rate of 100% required before clinical sample analysis. All testing procedures were conducted by trained laboratory personnel, and results were independently interpreted by two individuals before being recorded in an electronic data collection system. For samples with UBT results in the gray area (*n* = 12), participants were required to undergo retesting after a 4-week interval to obtain definitive results.

The entire UBT testing process adhered to the ISO 15189 quality management system requirements, with daily instrument calibration and recording of environmental temperature and humidity parameters (20°C–25°C, relative humidity ≤70%). The selection of clinical reference standards was based on the recommended levels for non-invasive testing methods outlined in the “Fifth National Consensus Report on the Management of *Helicobacter pylori* Infection” (Grade A evidence).

### Statistical analysis

Data analysis was performed using SPSS 26.0 (IBM Corporation). Categorical variables were expressed as frequencies and percentages, with inter-group comparisons made using McNemar’s test (paired samples) or χ² test (independent samples). Diagnostic consistency was assessed using Cohen’s Kappa coefficient, with Kappa values >0.75 indicating high consistency, 0.4–0.75 indicating moderate consistency, and <0.4 indicating low consistency. Diagnostic performance metrics (sensitivity, specificity, positive/negative predictive values) and their 95% confidence intervals (Clopper-Pearson method) were calculated using a 2 × 2 contingency table, with Youden’s index defined as sensitivity + specificity − 1. ROC curve analysis employed a non-parametric method to calculate the area under the curve (AUC), and differences in AUC between test and comparison reagents were evaluated using the DeLong test. The compliance rate for samples near the positive judgment threshold (CT values 37–42) was calculated using the exact binomial distribution to determine confidence intervals. All tests were two-sided, with *P* < 0.05 considered statistically significant.

## RESULTS

### Subjects

This study enrolled a total of 866 subjects. Following the screening process, 11 cases were excluded due to pre-experimentation, 43 due to screening failures, and 35 due to protocol deviations, resulting in 777 subjects eligible for statistical analysis. Specific details regarding the excluded cases are provided in Table S1. Among the final cohort of 777 subjects, there were 323 males (41.57%) and 454 females (58.43%), with a mean age of 46.34 years (age range: 18 to 75 years). The enrolled population included patients with gastric diseases, such as gastritis, peptic ulcers, and dyspepsia. A detailed demographic distribution is presented in Table 1.

**TABLE 1:** Distribution of study participants

Disease category	Cases (n)	Percentage (%)
Gastritis	152	19.56
Peptic ulcer disease	33	4.25
Dyspepsia	95	12.23
Asymptomatic suspected infection	107	13.77
Post-eradication therapy	53	6.82
Other symptoms (halitosis, abdominal pain, etc.)	233	29.99

### Positive detection rate

In this study, all subjects were collected and subsequently tested using the urea breath test (UBT), Cellrun PCR assay, and comparison reagents. The results indicated that the positive detection rate for UBT was 44.02%, for Cellrun PCR assay was 43.89%, and for comparison reagents was 44.14%. No statistically significant differences were observed in the positive detection rates between test and comparison reagents compared with UBT results (Table 2).

**TABLE 2:** Comparison of positive detection rates between two testing reagents and the urea breath test (UBT)

Method	Tested (*n*)	Positive (*n*)	Detection rate (%)	χ²	*P*-value
UBT	777	342	44.02	–^*a*^	–
Cellrun PCR assay	777	341	43.89	0.003	0.929
Comparison reagents	777	343	44.14	0.003	0.929

^*a*^ “–” indicates no statistically significant differences were observed.

### Consistency analysis

Using UBT results as a reference, the consistency between Cellrun PCR assay and UBT results was found to be excellent (Kappa = 0.9713, *P* < 0.001). Similarly, the consistency between comparison reagents and UBT results was also strong (Kappa = 0.966, *P* < 0.001; Table 3). In the comparison between test and comparison reagents, the positive agreement rate was 97.959% (95% CI 95.841%–99.176%), the negative agreement rate was 98.848% (95% CI 97.332%–99.625%), and the overall agreement rate was 98.456% (95% CI 97.318%–99.200%). The Kappa value was 0.9687 (95% CI 0.9511–0.9863), indicating substantial diagnostic consistency (Table 4).

**TABLE 3:** Consistency analysis of two testing reagents with UBT results

Detection method	UBT		Kappa	*P*
	Negative	Positive		
*n*	435	342		
Cellrun PCR assay, n (%)			0.971	<0.001
Negative	430 (98.9%)	6 (1.8%)		
Positive	5 (1.1%)	336 (98.2%)		
Comparison reagents, n (%)			0.966	<0.001
Negative	428 (98.4%)	6 (1.8%)		
Positive	7 (1.6%)	336 (98.2%)		

**TABLE 4:** Consistency analysis of results between cellrun PCR assay and comparison reagents

Detection method	Cellrun PCR assay		Kappa	*P*
	Negative	Positive		
*n*	436	341		
Comparison reagents, n (%)			0.969	<0.001
Negative	429 (98.4%)	5 (1.5%)		
Positive	7 (1.6%)	336 (98.5%)		

### Diagnostic efficacy analysis

**Fig 2:**
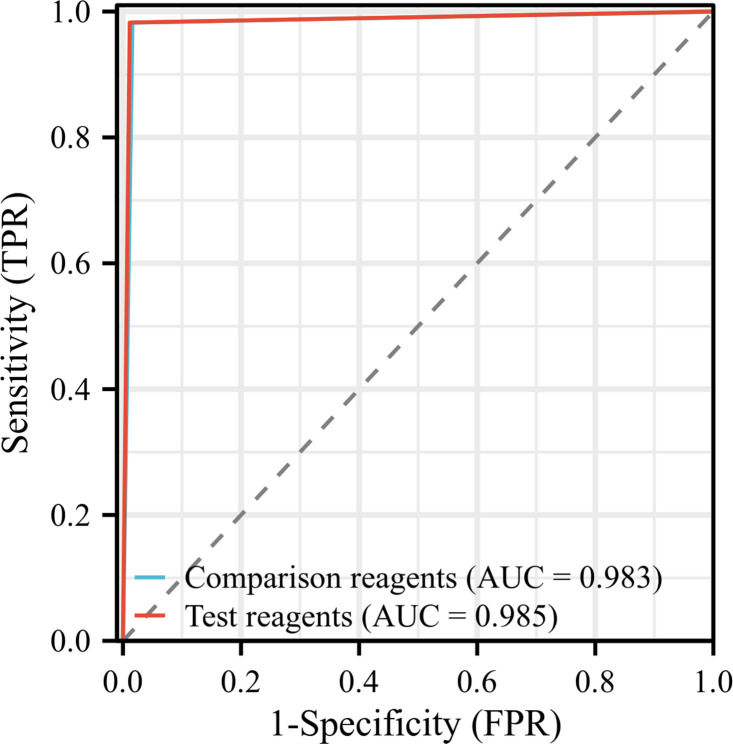
Diagnostic performance of Cellrun PCR assay and comparison reagents in predicting *Helicobacter pylori* Infection.

Using UBT results as a reference, the diagnostic sensitivity, specificity, accuracy, positive predictive value, negative predictive value, and Youden’s index for both test and comparison reagents are summarized in Table 5. Both reagents exhibited a sensitivity of 98.2%. The area under the curve (AUC) for Cellrun PCR assay was 0.985, which did not differ significantly from the AUC of 0.983 for comparison reagents (*P* = 0.614) (Fig. 2).

**TABLE 5:** Diagnostic performance analysis of cellrun PCR assay and comparison reagents

Predictor	AUC (95% CI)	Sensitivity	Specificity	Accuracy	PPV	NPV	Youden’s Index
Comparison reagents	0.983 (0.974–0.992)	0.982	0.984	0.983	0.980	0.986	0.966
Cellrun PCR assay	0.985 (0.977–0.994)	0.982	0.989	0.986	0.985	0.986	0.971

### Analysis of diagnostic efficacy for samples near the positive judgment threshold

Clinical trial samples for qualitative detection reagents should include a certain number of samples near the positive judgment threshold. In this trial, a total of 42 samples with weak positive results (CT values between 37 and 42) were included for comparison reagents, as detailed in Table S2. Cellrun PCR assay successfully detected 36 of these samples, resulting in a positive agreement rate of 85.714% (95% CI 71.461%–94.572%). These findings indicate a high level of consistency between Cellrun PCR assay and comparison reagents in distinguishing samples near the positive judgment threshold.

### Detection results of post-eradication therapy samples and interfering samples

**Fig 3:**
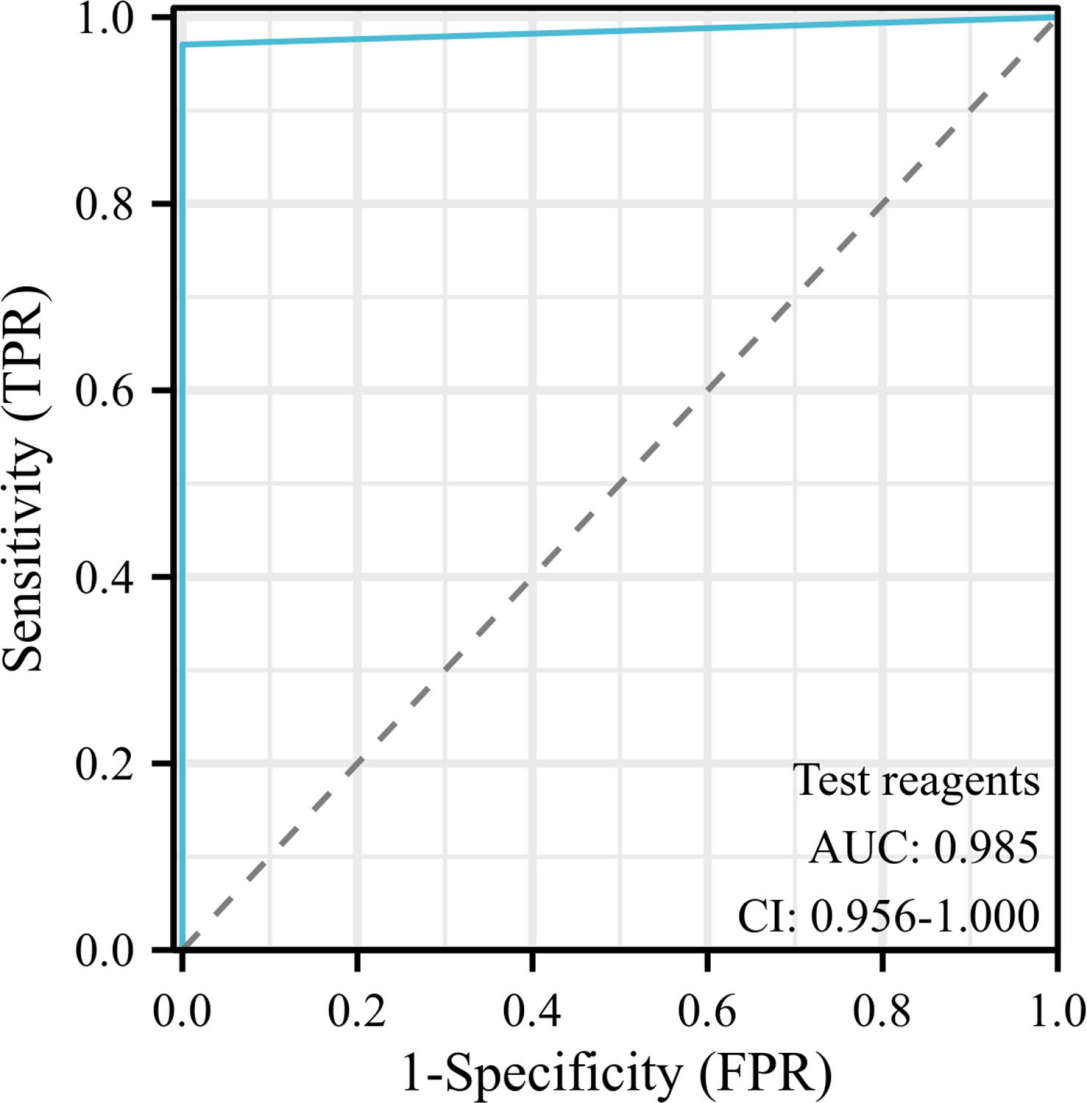
Diagnostic performance of Cellrun PCR assay in predicting *Helicobacter pylori* Infection in post-eradication samples.

This study included 53 subjects who underwent eradication therapy. In the stratified analysis of post-eradication patients, Cellrun PCR assay detected 18 out of 19 samples that were positive by UBT, yielding a sensitivity of 94.737% (95% CI 73.972%–99.867%). Among the 34 samples that were negative by UBT, Cellrun PCR assay identified 33, resulting in a specificity of 97.059% (95% CI 84.673%–99.926%) and an accuracy of 96.226% (95% CI 87.024%–99.540%). The Kappa value was 0.918 (95% CI 0.8065–1.000), and the AUC was 0.985 (Fig. 3).

Additionally, this study included 25 subjects with interfering samples, of which 21 were attributed to medications for treatment-related diarrhea (such as montmorillonite powder, intestinal anti-inflammatory agents, abdominal comfort, and compound phenylpiperidine), and four were due to false positives from routine fecal tests (including general characteristics, chemical examination, parasites, fecal culture, etc). The sensitivity of the Cellrun PCR assay results compared with UBT was 100.00% (95% CI 47.82%–100.00%), with a specificity of 100.00% (95% CI 83.16%–100.00%) and an accuracy of 100.00% (95% CI 86.281%–100.00%), yielding a Kappa value of 1.0000 (95% CI 1.0000–1.0000).

### Analysis of inconsistent results

A total of 11 samples exhibited inconsistent results between Cellrun PCR assay and UBT. Detailed information regarding these inconsistent samples is provided in Table S3. For samples 01100, 01131, 02154, 02157, 02159, and 02206, Cellrun PCR assay produced negative results, while UBT results were positive, potentially due to low bacterial concentrations leading to negative nucleic acid detection. Conversely, samples 01074, 02177, and 03143 yielded positive results with Cellrun PCR assay, while UBT results were negative, and comparison reagents were positive, likely reflecting discrepancies between the two testing methodologies. Samples 02143 and 02209 showed positive results from Cellrun PCR assay, whereas both UBT and comparator results were negative; these false positives may have resulted from detection errors near the threshold of the nucleic acid method.

There were 12 samples with inconsistent results between test and comparison reagents, with detailed information provided in Table S4. For samples 01046, 02155, 02193, and 04083, results from Cellrun PCR assay were negative, while comparator results were positive. Analysis of UBT results indicated consistency between Cellrun PCR assay and UBT, both being negative, while comparison reagents results were weakly positive, suggesting that comparison reagents’ performance may have led to false positive results. In samples 01100, 02157, and 02159, Cellrun PCR assay results were negative, while comparator results were positive, with UBT results also positive, potentially due to low bacterial concentrations and discrepancies in sample collection and processing methods, leading to false negative results. For samples 02143 and 02209, Cellrun PCR assay results were positive, while both comparator and UBT results were negative; given that the CT values for Cellrun PCR assay were in the weak positive range, these false positives may also be attributed to detection errors near the threshold of the nucleic acid method. Finally, for samples 03125, 04168, and 04192, Cellrun PCR assay results were positive, while comparator results were negative, and UBT results were positive, likely due to low *H. pylori* nucleic acid content in the samples failing to reach the detection limit of comparison reagents.

## DISCUSSION

*Helicobacter pylori* infection is a major pathogenic contributor to gastric cancer, with a prevalence rate of 38.0% in China, underscoring the importance of accurate diagnosis for the prevention and management of gastrointestinal diseases (32). While the urea breath test (UBT) has established itself as the gold standard due to its non-invasive nature and high sensitivity, its clinical utility is compromised by false negatives linked to proton pump inhibitor use, which can reduce sensitivity by 12%–33%, as well as ethical concerns associated with the use of the ^14^C isotope (33). Although fecal antigen detection has the advantages of low cost and simple operation, some components in feces may affect the affinity of antigen-antibody binding, thus easily causing false negatives (23). Molecular diagnostic techniques utilizing fecal samples, which directly detect bacterial DNA, present a promising alternative to address these limitations. Fecal PCR testing holds significant potential for vulnerable populations, such as children, the elderly, and individuals unsuitable for endoscopy. As a completely non-invasive method, it eliminates the risks associated with invasive procedures and avoids the radiation concerns of ^14^C-UBT, making it particularly suitable for pediatric and pregnant populations (17, 19). Its high sensitivity in detecting low bacterial loads (<10³ CFU/g) and coccoid forms ensures reliable diagnosis even in cases with minimal colonization (24). These advantages, coupled with its ability to detect drug resistance, position fecal PCR as an optimal screening tool for these specific patient groups. However, there remains a significant gap in multicenter, large-sample clinical validation to ensure consistency and reliability. This study enrolled 777 subjects to systematically evaluate the clinical efficacy of fecal PCR testing, thereby providing evidence-based support for optimizing non-invasive diagnostic strategies in response to the urgent need for precise diagnostics amid rising antibiotic resistance (34).

The findings of this study reveal a Kappa consistency coefficient of 0.9713 between fecal PCR and UBT, indicating a robust correlation between the two methods in diagnosing *H. pylori* infection. This strong correlation may be attributed to the high conservation of the 16S rRNA gene, which serves as a molecular marker for bacterial classification and effectively mitigates false negatives arising from antigenic variation (35). Additionally, the incorporation of a β-actin internal control system within Cellrun PCR assay significantly reduces interference from PCR inhibitors, such as humic acid present in fecal matrices, by monitoring the integrity of sample collection and nucleic acid extraction. Notably, in patients following eradication therapy, fecal PCR maintained a sensitivity of 94.7%, likely due to its capacity to detect residual DNA, whereas UBT, reliant on the metabolism of live bacteria, may yield false negatives (36). This differential performance provides complementary biomarkers for clinical efficacy assessment (37).

Both test and comparison reagents demonstrated area under the curve (AUC) values exceeding 0.98, with sensitivity and specificity surpassing 98%, confirming the high reliability of fecal PCR in detecting *H. pylori*. In comparison to traditional gastric mucosal biopsy, fecal sample testing avoids the invasive risks and sampling errors associated with endoscopy, making it particularly suitable for children, pregnant women, and patients with bleeding tendencies (38). Remarkably, Cellrun PCR assay achieved an 85.7% detection rate for weak positive samples (CT values 37–42), indicating superior low-burden detection capabilities compared to antigen-antibody dependent fecal antigen tests. This advantage may stem from an optimized nucleic acid purification system and a UDG enzyme contamination prevention system, which reduces the risk of aerosol contamination to below 0.1% by degrading uracil residues in prior amplification products, thereby significantly enhancing detection specificity (35). Furthermore, analysis of interfering samples revealed that Cellrun PCR assay maintained 100% sensitivity and specificity under conditions involving diarrhea-related medications and fecal occult blood interference, underscoring its broad clinical applicability. This characteristic aligns well with previous research emphasizing the importance of non-invasive testing in population screening (35).

Despite the high consistency observed between fecal PCR and UBT, the mechanisms underlying the 11 inconsistent samples warrant further investigation. Samples that tested positive by UBT but negative by PCR may indicate that colonizing bacteria in the gastric mucosa have not been shed into the intestinal tract, potentially related to variations in the expression of adhesins (such as BabA/BabB) among *H. pylori* strains, which can affect the adherence strength of bacteria to gastric epithelial cells and influence fecal shedding (39). Conversely, samples that were negative by UBT but positive by PCR may result from bacterial entry into a “dormant state” following antibiotic treatment, where metabolic activity is diminished, leading to UBT false negatives, while residual DNA remains detectable by PCR (16, 40). Moreover, comparison reagents exhibited a higher false positive rate in weak positive samples, potentially linked to probe design optimization or enhanced amplification efficiency. For instance, primers targeting conserved regions of the *H. pylori* 16S rRNA or urease genes may reduce detection failures due to genetic polymorphisms (41, 42). Additionally, the occurrence of false positives in certain samples with comparison reagents (e.g., weak positive sample 01046) may stem from cross-contamination or non-specific primer binding, while Cellrun PCR assay enhances specificity through optimized nucleic acid extraction processes (e.g., by incorporating steps to eliminate inhibitors) (43).

Although this study demonstrates the superiority of the novel *H. pylori* nucleic acid detection kit through multicenter and large-sample clinical validation, several limitations persist. First, the study primarily focused on a Chinese population; while the sample size is substantial, the external generalizability of the results requires further validation, particularly regarding performance in other geographic regions and diverse populations. Second, despite the excellent diagnostic sensitivity and specificity of Cellrun PCR assay, its ability to identify different genotypes of *H. pylori* necessitates further investigation. The genetic diversity of *H. pylori* may influence the diagnostic efficacy of PCR reagents. Future studies could explore the sensitivity differences of PCR detection across various *H. pylori* subtypes and strategies to enhance the reagent’s detection capabilities for low-concentration H. pylori samples. Additionally, integrating molecular biology with other diagnostic modalities (such as serological tests) may further improve the diagnostic accuracy and broader clinical applicability of *H. pylori* infection detection.

### Conclusion

This study provides robust multicenter clinical validation demonstrating that the fecal PCR test kit exhibits a high degree of concordance with the UBT and commercially available molecular diagnostic reagents in diagnosing *H. pylori*, showcasing notable diagnostic efficacy. The method retains high accuracy in samples obtained post-eradication therapy and in those potentially affected by interference. Nevertheless, the detection errors observed in borderline samples highlight the necessity for further optimization of nucleic acid extraction protocols and threshold settings. We recommend integrating molecular detection with metabolic testing strategies to enhance the precision of *H. pylori* diagnosis, particularly in samples with low bacterial loads or those near the detection threshold, as this approach may offer significant clinical value.

### Summary

#### Established knowledge of the subject

- *Helicobacter pylori* infection is a major cause of gastritis, peptic ulcers, and gastric cancer.
- The urea breath test (UBT) remains the gold standard for diagnosing active Hp infections but involves radiation exposure and equipment dependency.
- Non-invasive fecal molecular detection methods offer potential alternatives but require clinical validation for widespread adoption.

#### Significant and/or new findings of this study

- The novel fecal PCR assay (Cellrun, targeting 16S rRNA) demonstrated near-perfect diagnostic agreement with UBT (Kappa = 0.9713) and matched commercial kit (comparison reagents, Kappa = 0.966) in multicenter trials.
- High accuracy was achieved: sensitivity (98.2%) and specificity (AUC = 0.985) comparable to UBT, with 100% accuracy in interference-prone samples (κ = 1.0).
- Post-treatment performance remained robust (94.74% sensitivity, 97.06% specificity), supporting its clinical utility in monitoring Hp eradication.

### Study highlights

#### What is known

- *Helicobacter pylori* infection is strongly associated with gastritis, peptic ulcers, and gastric cancer.
- Urea breath test (UBT) remains the gold standard for active *H. pylori* diagnosis but carries radioactive risks and requires specialized equipment.
- Fecal molecular detection offers non-invasive potential but lacks sufficient multicenter clinical validation.

#### What is new here

- A novel 16S rRNA-targeted fecal PCR assay demonstrates near-perfect concordance with UBT (Kappa = 0.9713) and equivalence to commercial molecular diagnostics (AUC = 0.985 vs 0.983).
- The PCR assay achieves 98.2% sensitivity/specificity, maintains 85.7% agreement in weak-positive samples, and shows 94.74% sensitivity/97.06% specificity post-eradication therapy.
- Multicenter validation across 777 patients confirms 100% accuracy in interference samples and scalability for replacing radioactive/equipment-dependent methods.
- A novel 16S rRNA-targeted fecal PCR assay demonstrates near-perfect concordance with UBT (Kappa = 0.9713) and equivalence to commercial molecular diagnostics (AUC = 0.985 vs 0.983).

## Data Availability

The data sets used and/or analyzed during the current study are available from the corresponding author on reasonable request.

## SUPPLEMENTAL MATERIAL

Supplemental tables jcm.01762-25-s0001.docx Tables S1 to S3.

ASM does not own the copyrights to Supplemental Material that may be linked to, or accessed through, an article. The authors have granted ASM a non-exclusive, world-wide license to publish the Supplemental Material files. Please contact the corresponding author directly for reuse.
