# Supplementary material for: Clinical validation of a fecal PCR assay for Helicobacter pylori detection: multicenter comparative study with urea breath test and commercial molecular diagnostics
Source: J Clin Microbiol. 2026 Jul 27;64(8):e01762-25. doi: 10.1128/jcm.01762-25 (PMC13463709; doi:10.1128/jcm.01762-25)
Supplement: Supplementary file 1 [file jcm.01762-25-s0001.docx]

**Supplementary Table 1: Summary of excluded samples**

| No. | Screening Number | Enrollment Number | Reason for Exclusion |
| --- | --- | --- | --- |
| 1 | S01076 | 1067 | Sample size insufficient to complete subsequent testing, excluded per criterion 2 as determined by the investigator. |
| 2 | S01094 | 1087 | Sample preservation exceeded the allowable period, excluded per criterion 2. |
| 3 | S01132 | 1119 | Sample size insufficient to complete subsequent testing, excluded per criterion 2 as determined by the investigator. |
| 4 | S02046 | 2046 | Sample size insufficient to complete subsequent testing, excluded. |
| 5 | S02085 | 2085 | Negative quality control ineffective during Comparison Reagents testing; not retested in a timely manner as per instructions. Excluded. |
| 6 | S02086 | 2086 | Negative quality control ineffective during Comparison Reagents testing; not retested in a timely manner as per instructions. Excluded. |
| 7 | S02087 | 2087 | Negative quality control ineffective during Comparison Reagents testing; not retested in a timely manner as per instructions. Excluded. |
| 8 | S02088 | 2088 | Negative quality control ineffective during Comparison Reagents testing; not retested in a timely manner as per instructions. Excluded. |
| 9 | S02089 | 2089 | Negative quality control ineffective during Comparison Reagents testing; not retested in a timely manner as per instructions. Excluded. |
| 10 | S02090 | 2090 | Negative quality control ineffective during Comparison Reagents testing; not retested in a timely manner as per instructions. Excluded. |
| 11 | S02091 | 2091 | Negative quality control ineffective during Comparison Reagents testing; not retested in a timely manner as per instructions. Excluded. |
| 12 | S02092 | 2092 | Negative quality control ineffective during Comparison Reagents testing; not retested in a timely manner as per instructions. Excluded. |
| 13 | S02093 | 2093 | Negative quality control ineffective during Comparison Reagents testing; not retested in a timely manner as per instructions. Excluded. |
| 14 | S02094 | 2094 | Negative quality control ineffective during Comparison Reagents testing; not retested in a timely manner as per instructions. Excluded. |
| 15 | S02095 | 2095 | Negative quality control ineffective during Comparison Reagents testing; not retested in a timely manner as per instructions. Excluded. |
| 16 | S02096 | 2096 | Negative quality control ineffective during Comparison Reagents testing; not retested in a timely manner as per instructions. Excluded. |
| 17 | S02097 | 2097 | Negative quality control ineffective during Comparison Reagents testing; not retested in a timely manner as per instructions. Excluded. |
| 18 | S02098 | 2098 | Negative quality control ineffective during Comparison Reagents testing; not retested in a timely manner as per instructions. Excluded. |
| 19 | S02099 | 2099 | Negative quality control ineffective during Comparison Reagents testing; not retested in a timely manner as per instructions. Excluded. |
| 20 | S02100 | 2100 | Negative quality control ineffective during Comparison Reagents testing; not retested in a timely manner as per instructions. Excluded. |
| 21 | S02101 | 2101 | Negative quality control ineffective during Comparison Reagents testing; not retested in a timely manner as per instructions. Excluded. |
| 22 | S04180 | 4180 | Age did not meet enrollment criteria; excluded. |
| 23 | S04187 | 4187 | Age did not meet enrollment criteria; excluded. |
| 24 | S04190 | 4190 | Age did not meet enrollment criteria; excluded. |
| 25 | S04215 | 4214 | Duplicate enrollment; excluded. |
| 26 | S04216 | 4215 | Age did not meet enrollment criteria; excluded. |
| 27 | S04220 | 4219 | Duplicate enrollment; excluded. |
| 28 | S04221 | 4220 | Duplicate enrollment; excluded. |
| 29 | S04223 | 4222 | Duplicate enrollment; excluded. |
| 30 | S04224 | 4223 | Duplicate enrollment; excluded. |
| 31 | S04226 | 4225 | Age did not meet enrollment criteria; excluded. |
| 32 | S04231 | 4230 | Age did not meet enrollment criteria; excluded. |
| 33 | S04235 | 4234 | Duplicate enrollment; excluded. |
| 34 | S04236 | 4235 | Duplicate enrollment; excluded. |
| 35 | S04238 | 4237 | Duplicate enrollment; excluded. |

**Supplementary Table 2: Borderline positive samples near clinical cutoff**

| Screening Number | Enrollment Number | Test Reagents Detection Result | | | Comparison Reagents Detection Result | | | UBT Result |
| --- | --- | --- | --- | --- | --- | --- | --- | --- |
|  |  | Internal Control (CT) | H. pylori (CT) | Determination | Internal Control (CT) | H. pylori (CT) | Determination |  |
| S01059 | 01055 | 27.7126 | 32.3758 | Positive | 29.0036 | 38.5714 | Positive | Positive |
| S01075 | 01077 | 38.7394 | 36.6278 | Positive | 35.0887 | 37.5649 | Positive | Positive |
| S01084 | 01074 | 33.2349 | 38.3309 | Positive | 25.3964 | 37.8973 | Positive | Negative |
| S01103 | 01142 | 33.5972 | 36.7709 | Positive | 30.6453 | 37.1076 | Positive | Positive |
| S01112 | 01100 | 35.2855 | Undetermined | Negative | 30.2624 | 41.637 | Positive | Positive |
| S01142 | 01166 | 34.1297 | 37.1894 | Positive | 30.74 | 40.6071 | Positive | Positive |
| S01151 | 01128 | 34.5833 | 37.8811 | Positive | 31.9806 | 39.9514 | Positive | Positive |
| S01153 | 01135 | 35.2716 | 36.804 | Positive | 31.0676 | 39.0012 | Positive | Positive |
| S01175 | 01152 | 34.2633 | 39.044 | Positive | 30.8575 | 39.0756 | Positive | Positive |
| S01195 | 01175 | 36.0309 | 36.3719 | Positive | 31.392 | 37.3861 | Positive | Positive |
| S02007 | 02006 | 38.0030 | 35.8349 | Positive | 32.8312 | 40.2024 | Positive | Positive |
| S02012 | 02011 | 35.2685 | 33.9169 | Positive | 33.7642 | 38.1094 | Positive | Positive |
| S02015 | 02014 | 37.3907 | 34.9156 | Positive | 34.1638 | 37.1994 | Positive | Positive |
| S02023 | 02023 | 38.3045 | 35.4890 | Positive | 30.4815 | 37.3491 | Positive | Positive |
| S02045 | 02045 | 33.8758 | 35.1465 | Positive | 28.6417 | 41.1547 | Positive | Positive |
| S02051 | 02051 | 32.8654 | 34.6137 | Positive | 35.6510 | 40.1347 | Positive | Positive |
| S02054 | 02054 | 30.8836 | 37.1523 | Positive | 33.8724 | 39.5474 | Positive | Positive |
| S02055 | 02055 | 33.6773 | 37.6310 | Positive | 33.6790 | 38.2908 | Positive | Positive |
| S02056 | 02056 | 33.3617 | 37.0098 | Positive | 33.8025 | 37.5825 | Positive | Positive |
| S02058 | 02058 | 32.2073 | 35.2378 | Positive | 30.6645 | 37.6300 | Positive | Positive |
| S02103 | 02103 | 28.5452 | 32.0738 | Positive | 26.1039 | 39.6471 | Positive | Positive |
| S02156 | 02155 | 35.7209 | Undetermined | Negative | 31.1755 | 39.6502 | Positive | Negative |
| S02158 | 02157 | 34.1924 | Undetermined | Negative | 31.4274 | 37.4323 | Positive | Positive |
| S02160 | 02159 | 34.7208 | Undetermined | Negative | 31.9919 | 37.2210 | Positive | Positive |
| S02172 | 02171 | 35.9375 | 39.0261 | Positive | 31.4352 | 38.7819 | Positive | Positive |
| S02178 | 02177 | 25.0278 | 38.9420 | Positive | 29.2578 | 41.0849 | Positive | Negative |
| S02186 | 02185 | 35.0925 | 38.5921 | Positive | 31.2223 | 37.7728 | Positive | Positive |
| S02194 | 02193 | 35.0310 | Undetermined | Negative | 32.1506 | 38.8419 | Positive | Negative |
| S03095 | 03083 | 34.17 | 38.89 | Positive | 33.32 | 37.58 | Positive | Positive |
| S03120 | 03100 | 31.22 | 36.83 | Positive | 24.55 | 37.91 | Positive | Positive |
| S03176 | 03143 | 34 | 37.79 | Positive | 26.96 | 38.49 | Positive | Negative |
| S04075 | 04075 | 32.26 | 36.62 | Positive | 33.52 | 39.52 | Positive | Positive |
| S04083 | 04083 | 36.08 | Undetermined | Negative | 30.94 | 41.92 | Positive | Negative |
| S04115 | 04115 | 29.22 | 33.28 | Positive | 29.65 | 38.11 | Positive | Positive |
| S04124 | 04124 | 31.07 | 31.75 | Positive | 30.70 | 37.42 | Positive | Positive |
| S04128 | 04128 | 29.73 | 35.30 | Positive | 29.16 | 38.92 | Positive | Positive |
| S04129 | 04129 | 28.37 | 33.15 | Positive | 30.64 | 40.09 | Positive | Positive |
| S04164 | 04164 | 33.52 | 37.02 | Positive | 28.55 | 38.18 | Positive | Positive |
| S04179 | 04179 | 32.11 | 38.03 | Positive | 26.48 | 37.41 | Positive | Positive |
| S04185 | 04185 | 34.33 | 37.96 | Positive | 31.85 | 37.12 | Positive | Positive |
| S04188 | 04188 | 32.28 | 36.15 | Positive | 28.58 | 38.47 | Positive | Positive |
| S04199 | 04198 | 30.76 | 21.93 | Positive | 29.39 | 37.24 | Positive | Positive |
| S04219 | 04218 | 35.95 | 37.30 | Positive | 36.86 | 38.24 | Positive | Positive |
| S04243 | 04242 | 31.48 | 33.41 | Positive | 37.24 | 41.71 | Positive | Positive |

**Supplementary Table 3: Discordant results between test reagents and UBT**

| Screening Number | Enrollment Number | Test Reagents Detection Result | | | Comparison Reagents Detection Result | | | UBT Result |
| --- | --- | --- | --- | --- | --- | --- | --- | --- |
|  |  | Internal Control (CT) | H. pylori (CT) | Determination | Internal Control (CT) | H. pylori (CT) | Determination |  |
| 01 | 01100 | 35.2855 | Undetermined | Negative | 30.2624 | 41.637 | Positive | Positive |
| 01 | 01131 | 35.9878 | Undetermined | Negative | 31.1876 | Undetermined | Negative | Positive |
| 01 | 01074 | 33.2349 | 38.3309 | Positive | 25.3964 | 37.8973 | Positive | Negative |
| 02 | 02154 | 34.9385 | Undetermined | Negative | 28.5313 | Undetermined | Negative | Positive |
| 02 | 02157 | 34.1924 | Undetermined | Negative | 31.4274 | 37.4323 | Positive | Positive |
| 02 | 02159 | 34.7208 | Undetermined | Negative | 31.9919 | 37.2210 | Positive | Positive |
| 02 | 02206 | 35.5100 | Undetermined | Negative | 30.6121 | Undetermined | Negative | Positive |
| 02 | 02143 | 25.8737 | 38.2272 | Positive | 33.5915 | Undetermined | Negative | Negative |
| 02 | 02177 | 25.0278 | 38.9420 | Positive | 29.2578 | 41.0849 | Positive | Negative |
| 02 | 02209 | 34.8327 | 37.7036 | Positive | 28.5640 | Undetermined | Negative | Negative |
| 03 | 03143 | 34.0000 | 37.7900 | Positive | 26.9600 | 38.4900 | Positive | Negative |

**Supplementary Table 4: Discordant results between test and Comparison Reagents**

| Screening Number | Enrollment Number | Test Reagents Detection Result | | | Comparison Reagents Detection Result | | | UBT Result |
| --- | --- | --- | --- | --- | --- | --- | --- | --- |
|  |  | Internal Control (CT) | H. pylori (CT) | Determination | Internal Control (CT) | H. pylori (CT) | Determination |  |
| 01 | 01046 | 35.318 | Undetermined | Negative | 26.0122 | 34.7537 | Positive | Negative |
| 01 | 01100 | 35.2855 | Undetermined | Negative | 30.2624 | 41.637 | Positive | Positive |
| 02 | 02155 | 35.7209 | Undetermined | Negative | 31.1755 | 39.6502 | Positive | Negative |
| 02 | 02157 | 34.1924 | Undetermined | Negative | 31.4274 | 37.4323 | Positive | Positive |
| 02 | 02159 | 34.7208 | Undetermined | Negative | 31.9919 | 37.2210 | Positive | Positive |
| 02 | 02193 | 35.0310 | Undetermined | Negative | 32.1506 | 38.8419 | Positive | Negative |
| 04 | 04083 | 36.08 | Undetermined | Negative | 30.94 | 41.92 | Positive | Negative |
| 02 | 02143 | 25.8737 | 38.2272 | Positive | 33.5915 | Undetermined | Negative | Negative |
| 02 | 02209 | 34.8327 | 37.7036 | Positive | 28.5640 | Undetermined | Negative | Negative |
| 03 | 03125 | 33.5200 | 37.7900 | Positive | 28.2700 | Undetermined | Negative | Positive |
| 04 | 04186 | 31.29 | 31.07 | Positive | 29.00 | Undetermined | Negative | Positive |
| 04 | 04192 | 32.69 | 38.45 | Positive | 33.23 | Undetermined | Negative | Positive |
